# Letter to the Editor: Stemness-associated WNT3A and EDN3 as key regulators of tumor progression and immunotherapy efficacy in LUAD

**DOI:** 10.1097/JS9.0000000000003233

**Published:** 2025-08-21

**Authors:** Qianru Yang, Li Hou, Jing Wang

**Affiliations:** Department of Oncology and Hematology, Dongzhimen Hospital, Beijing University of Chinese Medicine, Beijing, China

*Dear Editor,*

We read with great interest the article entitled “Stemness-associated WNT3A and EDN3 as key regulators of tumor progression and immunotherapy efficacy in LUAD”[1]. The study provided valuable insights into the roles of WNT3A and EDN3 in lung adenocarcinoma (LUAD) and their association with immune evasion. However, several points need further discussion. The letter to the editor is compliant with the TITAN Guidelines 2025 – governing declaration and use of AI[2].

Firstly, although the authors observed a correlation between high WNT3A expression and reduced M0 macrophage levels, they did not investigate macrophage polarization or assess the functional states of other immune cell subsets, which are critical in mediating immunosuppression and resistance to therapy[3]. Furthermore, the reliance on bulk RNA-seq data and algorithms limited the ability to capture the functional state and spatial heterogeneity of immune cells within the tumor microenvironment[4]. To achieve a more comprehensive understanding of immune cell interactions and their impact on immunotherapy responses, future research should incorporate single-cell RNA sequencing or spatial transcriptomics.

Secondly, while EDN3 was found to significantly correlate with LUAD patient survival and diagnostic value, it showed no significant association with immunotherapy response. This limited discussion overlooks EDN3’s potential indirect roles. Notably, EDN3 is part of an eight-gene-signature prognostic model associated with cancer stemness and the tumor immune microenvironment[5]. Its stemness-related role warrants deeper investigation into its contribution to cancer stem cell (CSC) maintenance, metastatic potential, and resistance to therapy. Moreover, potential crosstalk with WNT3A and other oncogenic pathways remains unexplored, limiting mechanistic insights into LUAD progression.

Lastly, the authors validated the expression of WNT3A and EDN3 in limited cell lines using qPCR and Western blot, which may not adequately capture the diversity and complexity of LUAD tumors in patients[6]. Validating WNT3A and EDN3 as biomarkers or therapeutic targets necessitates multi-center studies with diverse patient cohorts to ensure equitable clinical applicability.

In summary, while the study presented compelling findings, future research should incorporate more advanced techniques and larger, more diverse clinical samples to fully explore the clinical implications of WNT3A and EDN3 in LUAD.

## Data Availability

All data are included in the article.
